# Planetary health in action: developing a heatwave vulnerability tool for primary care

**DOI:** 10.3399/BJGPO.2024.0089

**Published:** 2025-03-26

**Authors:** Karolina Griffiths, Paul Basso-Bert, Mireille Abraham, Elise Chin, Layana Caroupaye-Caroupin, Manal Ahikki, Emilie Agrech, Camille Debrock, Rim Sabri, Grégoire Mercier, François Carbonnel

**Affiliations:** 1 University of Montpellier, UMR UA11 INSERM - UM IDESP Institut Desbrest d'Épidémiologie et de Santé Publique Campus Santé, IURC, Montpellier, France; 2 University Department of General Practice, Faculty of Medicine of Montpellier-Nîmes, University of Montpellier, Montpellier, France; 3 General Practitioner, Primary Care Centre, Maison de Santé Pluriprofessionnelle Les Cevennes, Montpellier, France; 4 Ministère de la Transition écologique et de la Cohésion des territoires, Green Data for Health, Service de la Recherche et de l'Innovation, Commissariat général au développement durable (CGDD), Paris, France; 5 Public Health Department, Montpellier University Hospital, Montpellier, France; 6 The National Institute of Agronomy, Toulouse University, Toulouse, France; 7 Maison de santé pluriprofessionnelle universitaire Avicenne, Cabestany, France

**Keywords:** data, heatwaves, environment, climate, primary health care

## Abstract

**Background:**

Heatwaves are becoming longer and more frequent. Despite the availability of open environmental data, little is operable and formatted for primary care use.

**Aim:**

To create a user-friendly online mapping tool to assess the vulnerability of communities to heatwaves for use by primary care practitioners. This study questioned what knowledge needed to be deployed, who needed to participate, and how the knowledge should be shared.

**Design & setting:**

A participatory action-research project based on knowledge mobilisation (KM) in France, as part of the Green Data for Health Challenge.

**Method:**

Knowledge was summarised on the factors most affecting heatwave vulnerability in a collaborative process, enabling a consensus on data variables and mobilised content for the online tool. Purposive sampling included primary care stakeholders with Regional Health Agencies (Agence Régionale de Santé; ARS), Public Health France (Santé Publique France; SPF), and data scientists.

**Results:**

Nineteen participants took part in 10 co-construction meetings, a brainstorming carousel strategy, and five weekly co-creation meetings between December 2022 and June 2023. The heatwave vulnerability variable was constructed using surface temperature, social deprivation, vegetation coverage, and presence of air-conditioning equipment. Identified experts mobilised data on the national composite indicator at the communal level for heatwave morbidity.

**Conclusion:**

There is no standard platform for sharing environmental data in France. This co-creation study offers a new approach to incorporating environmental data on heatwaves into primary care consultations. We demonstrate the importance of KM in primary care to bridge the research–practice gap. Integrating primary care records with environmental data may promote broader applications for planetary health research.

## How this fits in

Heatwaves are becoming longer and more frequent, and the cause of an estimated 33 000 deaths in France in 2014–2022. Primary care clinicians must be aware of the risks, yet heatwave vulnerability tools are often directed at urban planners. We present an action-research project that developed an online tool for GPs in France to assess heatwave vulnerability. This is an example of applying environmental data for clinical primary care, opening the door for other uses for planetary health.

## Introduction

Planetary health can be defined as *‘the health of human civilization and the state of the natural systems on which it depends'* in a transdisciplinary, solutions-oriented social movement.^1^ An estimated 23% of global deaths can be attributed to the environment.^2^ The Intergovernmental Panel on Climate Change (IPCC) estimates that the average global temperature increased by 1.09°C, comparing 2011–2020 with the pre-industrial period (1850–1900).^3^ Regardless of emission scenarios, global warming could reach+1.5°C by the 2030s. This overall temperature increase results in significant local temperature differences. In Europe, the average surface temperature has risen by 2.2°C since the pre-industrial era (1850–1900), twice the global average. This leads to longer, more intense, and more frequent heatwaves, alongside extreme weather events such as droughts and wildfires.^3^ A heatwave is a prolonged period of exceptionally hot weather compared with the typical conditions for that time of year, often accompanied by elevated humidity. Heatwaves caused an estimated additional 33 000 deaths in France during the summers of 2014–2022.^4–6^ Three heatwaves occurred in 2022, leading to heatwave alerts in 69 French departments, covering 77.8% of the population.

The World Health Organization (WHO) and IPCC emphasise the need for a holistic and cross-sectoral approach to address climate change challenges.^3^ The Physiological Society consensus statement, *Roadmap for Global Heat Resilience*, reinforces the need for enhanced collaboration with transdisciplinary approaches by creating networks to facilitate the exchange of ideas and practices for heat resilience and facilitate knowledge sharing.^7^ A first step is to identify vulnerable populations and target local-scale interventions, with an important role for local actors including healthcare professionals.^8^ There is a lack of knowledge among doctors regarding planetary health, the effects of climate change and heat.^9^ Sixty-eight per cent of surveyed French GPs were unaware of heatwave recommendations.^10^ Only 20% of doctors reported monitoring high-risk patients more frequently during heatwaves. Despite 93% of GPs believing they play an important role in informing their patients about environmental risks, only 55% felt capable.^11^

How can we apply environmental data to primary health care? Huge amounts of environmental data are already available, often with easy and free access. Internationally, various databases exist: the New Jersey Environmental Public Health Tracking (EPHT) Network in the US^12^ and, in Europe, the Environmental and Health Information System.^13^ There are numerous public open databases for the French territory such as Public Health France GeoData 'GEODES'^14^ and Interregional health informatics system 'SIRSé'^15^ However, these datasets are often independent and isolated, leading to a lack of standardisation and interoperability, and not intended for use by clinicians.^16,17^ These challenges in using environmental data led to the development of an initiative to share environmental data for health, known as the Green Data for Health, led by the fourth National Environmental Health Plan of the French government.^18^ We describe this knowledge mobilisation (KM) process: how to optimise the use of knowledge, and how to disseminate and apply this research to concrete action in practice.^19^

The aim of this study was to create a user-friendly online mapping tool to assess the vulnerability of communities to heatwaves for use by primary care practitioners.

## Method

### Context

The action-research took place December 2022–June 2023 in France. The study was based on participatory methods in KM^19,20^ within the context of the 'Green Data for Health Challenge', launched by the French government in October 2022 to produce new tools to better mobilise environmental data for health use.^18^ The challenge included a national call to action survey, from which our project was selected. The study is reported in line with the checklist for assessing quality in action research.^21^

### Recruitment

The focus was on co-design, emphasising collective decision-making in collaboration with end users. The project stems from a 'bottom-up' model: the initial idea was submitted by the GP and public health team (KG, PBB, FC, GM), who are both researchers and participants in this participatory project. Early engagement from academics and clinical stakeholders are prerequisites to achieve impact in KM.^22^ The initial researchers and the facilitator were all experienced in co-creation. Further participants were included during the iterative process. Maximum variation purposive sampling involved selecting participants with different roles to actively contribute to the design process, ensuring variability between primary care, public health, data scientist, and technical roles. Workshops were virtual video meetings considering the participants' geographical locations.

### Preparation

A literature review in December 2022 using PubMed and MEDLINE on heatwaves, morbidity and mortality, and risk factors guided the co-creation workshops (Supplementary Data). The carousel strategy,^23^ kickstarted the process and identified four questions to address (Table 1).

**Table 1.** Four questions identified to address during knowledge mobilisation (KM)

What data are available to measure the impact of heatwaves on human morbidity and mortality pertinent to primary care use?
Which indicators are available to model the vulnerability of the French population to heatwaves?
What team members and roles are needed to co-create this tool online?
What platform is best to share this tool to reach primary care stakeholders in France and how do we evaluate the use?

### Action

There was a constraint of a 2-month 'tool production' window April–May 2023. We incorporated a creative co-design in an iterative, collaborative process to generate solutions and design the mapping tool. Weekly co-creation workshops permitted iterative feedback to define the format and content of the online tool within this short timeframe. The challenge facilitator provided momentum and initiated interdisciplinary contacts.

National data sources from metropolitan France were used. The commune is the smallest administrative unit, usually covering a small area (mean 14.9 km^2^) but with a variable population size, ranging from small (around 500) to very large in cities. Population size in each of the 95 metropolitan departments ranges from 76 000 to >2 million.^24^

### Data collection and analysis of the knowledge mobilisation process

Data collection included online transcriptions and field notes from 10 co-construction meetings, a group brainstorming carousel session, and five co-creation workshops, totalling 23 hours of observation. The co-researchers used a 'memoing' technique, operational memos described observations, rationale and actions, and analytical memos explored hypotheses and relationships.^25^ Summaries were circulated using an online data-sharing forum called Slack, allowing modifications and comments. The co-researchers (as both participants and researchers) maintained a reflexive position.

## Results

Table 2 demonstrates participant characteristics. There was overlap between the roles, for example, GP researcher, clinical and academic roles, and also future user. This co-creation group addressed four questions in the development process. Responses are discussed below with examples from the data collected from the workshops (Table 3).

**Table 2.** Participant characteristics

Role	Sex	Age range, years
Practice coordinator + future user	M	30–44
GP, clinical and academic roles, + future user	M F F	30–44 30–44 30–44
GP trainees + future user	F M	18–30 18–30
Public health doctors	M	30–44
Data scientist	F F F F	18–30 18–30 18–30 18–30
Data analyst	F F	18–30 18–30
Facilitator, epidemiologist	M	30–44
Geometrician	F	18–30
Regional health department sanitary engineer and project leader	F	45–60
Project leader for the national mapping tool AtlasSanté	F	30–44
Health geographer	F M	30–44 30–44

**Table 3.** Details of co-creation workshops

Workshop	Time	Discussions and tasks:
Carrousel	4 hours	In four parts: Pitch: define objectives and questions to address Subject pertinence and generalisability Method feasibility: identify the needs, tools, software, personnel Data availability and data quality: identifying potential issues in advance
Workshop 1	1 hour	Discussion on indicators, source Decision to use France communes as mapping scale
Workshop 2	1 hour	Temperature data extraction, decision to use satellite data Inclusion of data on buildings equipped with air conditioners How to deal with missing data from the sociological variable FDep? How to take into account vegetation land cover?
Workshop 3	1 hour	Satellite temperature data extraction
Workshop 4	1 hour	CORINE Land Cover, calculation of Z scores
Workshop 5	1 hour	Calculation of final score

CORINE = Coordination of Information on the Environment

### What data are available to measure the impact of heatwaves on human morbidity and mortality pertinent to primary care use?

Quantifying heat-related mortality and morbidity is complex. Because heat has multiple effects on health, it is not possible to identify heat-attributable deaths in real-time based on a predefined single cause of death.^4^ When mapping small numbers of cases on small geographic units there is a risk of overestimating spatial heterogeneity, leading to significant variations induced by statistical noise. Death certificates are not always reliable. Public Health France monitors the impact of heatwaves and provides mortality data at the departmental level (GEODES).^14^ Mortality data at the communal level are not available.

**Figure 1.**
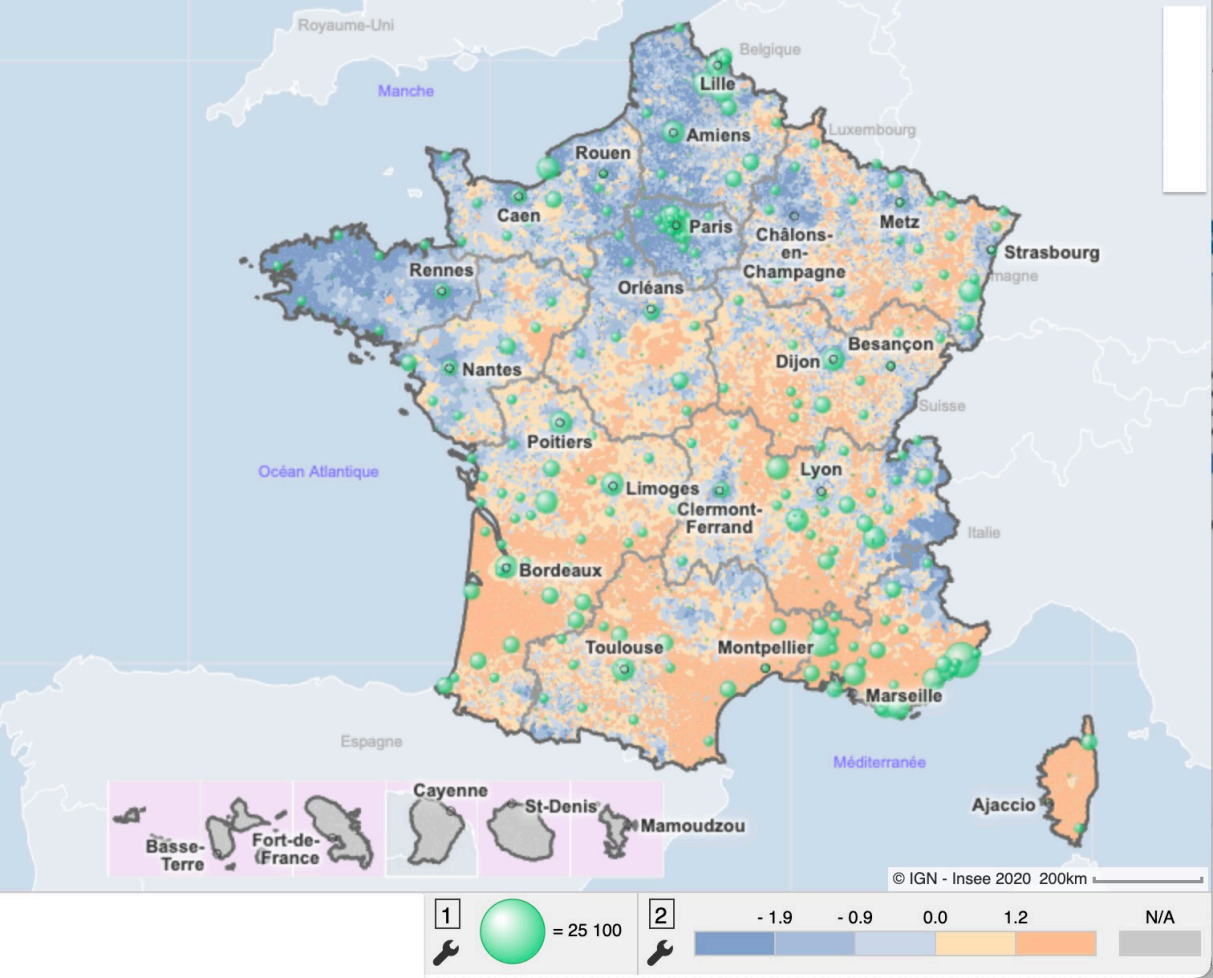
Heatwave vulnerability map. 1 = iCanicule indicator: number of emergency department and emergency GP visits for heatwave morbidity, which is based on the number of emergency department visits and emergency GP visits for hyperthermia, heatstroke, dehydration, and hyponatremia. Canicule is the French term for heatwave. 2 = Heatwave vulnerability index: composite indicator of four variables (FDep, CORINE Land cover rate, average summer surface temperature, rate of equipment of residential buildings with air conditioning)

The discussions identified the lack of heatwave-related morbidity data. Therefore, a Public Health France epidemiologist was recruited to the following workshop. We identified the public health need for a national composite indicator at the communal level for heatwave morbidity: based on the number of emergency department visits and emergency GP visits for hyperthermia, heatstroke, dehydration, and hyponatremia (Figure 1). This 'iCanicule' indicator was produced by Public Health France.

### Which indicators are available to model the vulnerability of the French population to heatwaves?

A literature review elicited numerous factors that influence population vulnerability to heatwaves. The discussions reached a consensus that apart from temperature, four further factors should be included: urbanisation, vegetation coverage, air conditioning, and socioeconomic status (Table 4). The choice of a communal-level resolution was influenced by data availability. The construction of the indicators is explained in further detail in the data paper.^26^

**Table 4.** Variables included in the vulnerability score that can influence heatwave-related morbidity and mortality, All variables at the 'commune' level, the smallest administrative unit in France

Variable	Source	Date stamp
**French Deprivation Index (FDep)** A score that includes information on: unemployment rates and the percentage of blue-collar workers in the active population aged 15–64 years, the percentage of high-school graduates in the population aged ≥15 years, and the median household income	Inserm, CépiDC	2015^a^
Mean daily surface temperature	Copernicus Climate Data Store	Mean June–August 2022
Percentage of primary residences equipped with an air-conditioning unit	BDNB National Building Database from Insee	2022^a^
**CORINE Land cover** This database, created by the European Environment Agency, divides each 25-hectare square into 44 land use classes using satellite data and aerial photos	European Environment Agency’s Copernicus Land Monitoring Service	2018^a^

^a^The latest available year was included. BDNB = Base de Données Nationale des Bâtiments (National Buildings Database). CépiDC = Le Centre d'épidémiologie sur les causes médicales de décès (Center for Epidemiology on Medical Causes of Death). CORINE = Coordination of Information on the Environment. Insee: Institut national de la statistique et des études économiques (National Institute of Statistics and Economic Studies), Inserm = Institut national de la santé et de la recherche médicale (National Institute of Health and Medical Research)

There are numerous sources of land and air temperatures. We reached a consensus to use satellite-related daily mean temperature at the communal level.^27^

There is a protective effect of vegetation.^28^ The integration of the proportion of artificial and non-vegetated surfaces (CORINE [Coordination of Information on the Environment] Land Cover) incorporates this effect in the vulnerability index calculation.^29^

Access to air-conditioned space is linked to heatwave vulnerability.^30^ French data on the presence of an air-conditioning unit is available by commune.^31^

The link between heatwaves and social deprivation is known.^32,33^ Data on unemployment rates, education attainment, and poverty rates are available at the communal level.^15^ We used the French Deprivation Index (FDep), correlated with the total mortality rate.^34^

### What team members and roles are needed to co-create this tool online?

An iterative approach was used: the co-researchers recognised the need to break the project down into multiple stages and recruit specific roles. Purposive sampling included data scientists, geometricians, and data analysts (Table 2). Interactions took place through 1-hour weekly remote meetings over 5 weeks using the software program Slack. At each meeting there was a systematic sharing of progress before setting the goals to be achieved for the following week. Each team member was assigned a specific task to complete in the coming week on a voluntary basis (Table 3).

### What platform is best to share this tool to reach primary care stakeholders in France and how do we evaluate the use?

There is no standardised platform for online tools for primary care in France. The open-access heat vulnerability indicator map and iCanicule indicator was made available on the website SIRSé.fr on 5 June 2023.^15^ The link was shared via GP networks. Initial feedback was obtained from one focus group of users in March 2024 to test the site with 10 GPs from two multidisciplinary practices in the south of France. It was facilitated by one of the authors (KG) with a simple interview guide with open-ended questions about their experiences with managing heat-related illnesses and opinion of the mapping tool and relevance to medical practice. Feedback included that the site at a national level was informative, and coherent to compare between departments, but less useful at the local scale. The map scale was only at communal level, and *'needed to be more precise, at the street le*vel'. The GPs were interested in more precise morbidity and mortality data, which are not yet available, and the relation to other clinical data. Other doctors suggested that mapping other environmental variables, not just heatwaves and heatwave vulnerability, could be useful to better understand the specific risks in each territory. Further in-depth interviews and focus groups and formal qualitative analysis could explore the impact of this mapping tool on medical practice. Further quantitative evaluation will include the number of users.

## Discussion

### Summary

An online mapping tool for heatwave vulnerability^15^ was successfully developed that incorporates variables for surface temperature, social deprivation, vegetation coverage, and air-conditioning equipment.^26^ It was developed with and for primary care stakeholders in a collaborative process with Regional Health Agencies (Agence Régionale de Santé; ARS), Public Health France (Santé Publique France; SPF), and data scientists. Knowledge was synthetised on the factors most affecting heatwave vulnerability, enabling a consensus for the choice of indicators and mobilised content for the online tool.^15^

This co-creation study offers a new approach to including environmental data on heatwaves into the primary care consultation, going beyond sparse existing educational interventions for patients and primary care professionals. This study questioned what knowledge needed to be deployed, who needed to participate, and how the knowledge should be shared. Responses to these questions were elicited through co-creation workshops and online discussions.

### Strengths and limitations

Mapping tools provide a quick visual summary to improve understanding of heatwave vulnerability. Our tool enables comparisons at a small communal scale. Owing to the iterative process and purposive sampling, the co-construction team was varied, with data science and cartography experts. KM encouraged collaboration, flexibility and pragmatism, and objectives evolved according to methodological constraints. The difficulties in KM are well-known, notably the challenges and time constraints in bringing stakeholders together.^22^ All workshops were online, however facilitation and online software programs maintained momentum and encouraged team participation.

The research methodology chosen was the most feasible with the time constraints and resources available. Other possible methods include systematic literature review or Delphi consensus.

### Comparison with existing literature

Our heatwave vulnerability tool was based on previous studies using a mapping tool.^35^ The City of New York has published an online interactive index for each neighbourhood including the average daily temperature during heatwaves, access to air-conditioned space, the rate of green spaces, the percentage of inhabitants living below the threshold of poverty, and the percentage of Black residents.^36^

Population density is a significant exposure factor. It is directly correlated with urbanisation and the 'urban heat island' effect.^37^ Surface temperature, theoretically higher in more urbanised areas, directly represents this phenomenon. A European *Lancet* report identifies the following two indicators: (1) vulnerability to heat exposure that includes: percentage of adults aged >65 years, who live in urban areas and have a chronic disease (diabetes, cardiovascular, respiratory or kidney disease); and (2) exposure of vulnerable populations to heatwaves, defined as people aged >65 years and infants<1 year.^38^

The final selection of indicators was based on data availability. We did not integrate individual risk factors for heat mortality identified by epidemiological studies.^39–41^ For example, the standardised prevalence of people treated for chronic conditions is only published at the departmental level. Additionally, data on ethnicity and race cannot be collected in France.^42^ We constructed a composite index using equal weights for each indicator, as in previous studies modelling heatwave vulnerability.^36^ However, this does not allow indicators to be weighted to their probable impact, which requires additional study.

We have demonstrated successful KM in primary care. A key facilitator of KM is integrated care records; updating local information in real-time into primary care records, such as the UK Clinical Practice Research Datalink (CPRD).^19^ A French equivalent, Platform for Data in Primary Care (P4DP), is currently being deployed in French primary care. The next step is to integrate inter-operable environmental variables into care records, using clinical data alongside the heatwave vulnerability index.

### Implications for research and practice

Harnessing a diverse range of environmental data, which are not only on heatwave vulnerability, could guide primary care professionals and patients. Implications for primary care practice include enhancing preventive care, personalising risk assessments, and targeted interventions to combat health disparities and improving community health interventions, education, and advocacy.^43^ We demonstrate the importance of KM in primary care to bridge the research–practice gap. However, future studies are needed to improve spatial resolution. Primary care centres are grouped into territorial areas in France and are not aligned to other communal maps. Presenting the information at a politically relevant level would guide healthcare professionals to perform environmental health territorial diagnostics and improve patient interventions. Further studies are needed to integrate environmental data directly into care records, allowing healthcare professionals to better understand local heatwave vulnerability and prevent heatwave-associated morbidity and mortality.

## Data

The datasets used and/or analysed during the current study are available from the identified sources or from the corresponding author on reasonable request
